# Confined Polysulfides in N-Doped 3D-CNTs Network for High Performance Lithium-Sulfur Batteries

**DOI:** 10.3390/ma14206131

**Published:** 2021-10-15

**Authors:** Donghuang Wang, Aijun Zhou, Zhujun Yao, Xinhui Xia, Yongqi Zhang

**Affiliations:** 1 Yangtze Delta Region Institute (Huzhou), University of Electronic Science and Technology of China, Huzhou 313001, China; wdh@csj.uestc.edu.cn (D.W.); zhouaj0823@163.com (A.Z.); helloxxh@zju.edu.cn (X.X.); 2 School of Materials and Energy, University of Electronic Science and Technology of China, Chengdu 611731, China; 3 School of Materials Science and Engineering, Zhejiang Sci-Tech University, Hangzhou 310018, China; yaozj@zstu.edu.cn; 4 State Key Laboratory of Silicon Materials, Key Laboratory of Advanced Materials and Applications for Batteries of Zhejiang Province, School of Materials Science and Engineering, Zhejiang University, Hangzhou 310027, China; 5 Institute of Fundamental and Frontier Sciences, University of Electronic Science and Technology of China, Chengdu 611731, China

**Keywords:** lithium polysulfides, CNTs network, N-doping, lithium-sulfur battery

## Abstract

Improving the utilization efficiency of active materials and suppressing the dissolution of lithium polysulfides into the electrolyte are very critical for development of high-performance lithium-sulfur batteries. Herein, a novel strategy is proposed to construct a three-dimensional (3D) N-doped carbon nanotubes (CNTs) networks to support lithium polysulfides (3D-NCNT-Li_2_S_6_) as a binder-free cathode for high-performance lithium-sulfur batteries. The 3D N-doped CNTs networks not only provide a conductive porous 3D architecture for facilitating fast ion and electron transport but also create void spaces and porous channels for accommodating active sulfur. In addition, lithium polysulfides can be effectively confined among the networks through the chemical bond between Li and N. Owing to the synergetic effect of the physical and chemical confinement for the polysulfides dissolution, the 3D-NCNT-Li_2_S_6_ cathodes exhibit enhanced charge capacity and cyclic stability with lower polarization and faster redox reaction kinetics. With an initial discharge capacity of 924.8 mAh g^−1^ at 1 C, the discharge capacity can still maintain 525.1 mAh g^−1^ after 200 cycles, which is better than that of its counterparts.

## 1. Introduction

There has been a strong demand of late for developing safe and cheap cathode materials with high energy density of rechargeable lithium batteries for many applications, such as portable electronic devices, electric vehicles, and the grid storage of electricity [1,2,3,4,5,6,7,8]. Among the various alternative energy storage systems, the lithium-sulfur (Li-S) batteries are considered as one of the most promising candidates for next-generation energy storage devices, owing to the extremely high theoretical specific capacity (1672 mAh g^−1^) of sulfur [9,10]. Additionally, sulfur is a cheap, low-toxic and abundant resource, which makes Li-S batteries a particularly low-cost and attractive energy storage technology [11,12]. However, Li-S batteries still hindered by the following critical challenges [13,14,15]. Firstly, the element sulfur is insulating with the high resistance of about 5 × 10^−30^ S cm^−1^, resulting into a low utilization of active materials and a large internal resistance and polarization of the cathode [16,17]. Secondly, a volume expansion of about 80% exists into electrochemical conversion of sulfur (density of 2.03 g cm^−3^) to Li_2_S (density of 1.66 g cm^−3^), giving rise to structural and morphological destruction and poor columbic efficiency and rate capacity [18,19]. Thirdly, lithium polysulfides (Li_2_S*_x_*, 3 ≤ *x* ≤ 8) intermediates formed during the charge/discharge progress are soluble in the electrolytes, leading to a loss of active materials, self-discharge and capacity fading during extended cycling [20,21].

To address these thorny problems, various strategies are developed, including encapsulation of sulfur into conductive host materials, embedment of interlayers and optimization of the electrolytes or additives [22,23,24,25]. A relatively successful approach is to design carbon-based hosts (e.g., mesoporous carbon, hollow carbon nanofiber, graphene and carbon nanotubes) [26,27,28,29,30]. Typically, combination sulfur with carbon nanotubes are regarded as an effective way for construction of high-performance sulfur cathodes, which could improve both electron and ion transfer and accommodate volume changes during cycling [31,32,33]. However, only physical confinement is still insufficient to solve the polysulfide shuttle problem because of the open holes and weak interactions between carbon and polysulfides. Additionally, significant research effort has been made towards chemical confinement of polysulfide intermediates by heteroatom doping, especially nitrogen doping [34,35,36,37]. First-principle calculation and surface analysis further proved that polar-polar interaction and Lewis acid-base interaction with polysulfides are strong and stable [38,39]. Therefore, the nitrogen doping carbon nanotubes (CNTs) may have a significant effect on suppressing the diffusion of polysulfides and realize high-performance of lithium-sulfur batteries. Undoubtedly, it is still necessary to develop a novel and effective methodology for construction three-dimensional architecture with N-doping as high-loading sulfur carriers and take full advantage of the interactions to suppress polysulfides diffusion for realization of high-energy and commercially viable Li-S batteries.

Herein, we develop a novel strategy to construct an integrated cathode by confining polysulfides into three-dimensional N-doped CNTs networks (3D-NCNT-Li_2_S_6_). The N-doped CNTs have constructed a 3D conductive networks, which not only provide conductive path for electron and ion transfer but also create large amounts of porous channels and void spaces to for a high sulfur loading. In addition, the physical and chemical confinement have a synergetic effect on suppress the polysulfides dissolution into the electrolyte. The cathodes demonstrate enhanced performance with lower polarization, better cycling stability and superior high-rate performance.

## 2. Materials and Methods

### 2.1. Preparation of 3D-NCNT-Li_2_S_6_ Composite

As reported previously, the three-dimensional CNTs network was grown on the carbon cloth (3D-CNT) by a chemical vapor deposition (CVD) method. Typically, the carbon cloth (CC) was firstly immersed in nickel nitrate ethanol solution for 5 min and dried under 60 °C. Then the prepared carbon cloth was thermally treated at 600 °C in a mixed-gas atmosphere of 60 sccm Ar + 5 sccm H_2_ for 30 min. And C_2_H_4_ gas was introduced as a carbon source with a flow rate of 10 sccm for another 30 min to obtain 3D-CNT. The 3D-CNT was further doped nitrogen through the treatment at 550 °C with NH_3_ (30 sccm) for 1 h to form the 3D-NCNT.

0.5 M Li_2_S_6_ solution was prepared by mixing sulfur and Li_2_S at a molar ratio of 5:1 in an appropriate amount of 1,2-dimethoxyethane and 1,3-dioxolane (DME and DOL). To fabricate 3D-NCNT-Li_2_S_6_ cathode, 10 μL polysulfide solution was added into 3D -NCNT that was cut into 0.5 × 0.5 cm^2^ and dried in argon atmosphere. For comparison, the 3D -CNT- Li_2_S_6_ cathode was synthesized in the same way without NH_3_ treatment.

### 2.2. Materials Characterizations

The morphologies of these samples were investigated by scanning electron microscope (SEM, Hitachi S-4800, Tokyo, Japan) and the elemental composition and mapping were using EDS on the SEM. Raman measurements were performed on a Jobin Yvon Labor Raman HR-800 (Paris, France) using a 532 nm diode-pumped solid-state laser after the samples were sealed in a chamber with a glass window in glove box. Surface groups and bond of samples were characterized by an X-ray photoelectron spectroscopy (XPS, ESCAL 220i-XL, Waltham, MA, USA), using Al Kα X-ray radiation source in a base pressure of 10^−7^ Pa.

### 2.3. Electrochemical Measurements

The CR2025 coin cells were assembled with the 3D-CNT-Li_2_S_6_ and 3D-NCNT-Li_2_S_6_ composites as the working electrode, pure lithium foil as the counter electrode and cellgard 2400 as the separator. The cells were assembled in an argon filled glove box with O_2_ and H_2_O concentration below 0.1 ppm and 1 mol L^−1^ lithium bis (trifluoromethanesulfonyl) imide (LiTFSI) in 1,3-dioxolane (DOL) and 1,2-dimethoxyethane (DME) (1: 1 by volume) with 1 wt.% lithium nitrate (LiNO_3_) as an additive was used as the electrolyte. The areal mass loading of S on the electrodes is about 3.84 mg cm^−2^. All the specific capacities and current densities of cells were calculated on the basis of the mass of sulfur. The galvanostatic measurements were evaluated by the LAND battery test system in potential range from 1.7 to 2.8 V. The cells were first discharged to 1.7 V and then the cycle number was counted. Cyclic voltammetry (CV) tests were carried out on a CHI660E electrochemical workstation (Shanghai, China) at a scan rate of 0.1 mV s^−1^. All the electrochemical tests were conducted at room temperature.

## 3. Results and Discussion

**Figure 1:**
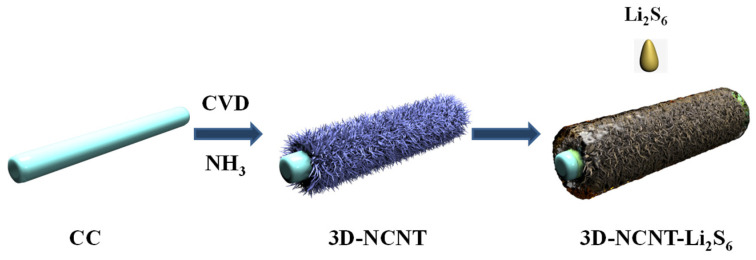
Illustration of synthesis 3D-NCNT-Li_2_S_6_ composite.

The fabrication of 3D-NCNT-Li_2_S_6_ is schematically illustrated in Figure 1. Firstly, the three-dimensional CNTs network was grown on the carbon cloth (3D-CNT) by a CVD method. Then, the nitrogen-doping is introduced into the 3D-CNT through NH_3_ treatment to form the 3D-NCNT. Finally, the Li_2_S_6_ solution was added into the 3D-NCNT to obtain the 3D-NCNT-Li_2_S_6_ cathode.

### 3.1. Structural Characterization

**Figure 2:**
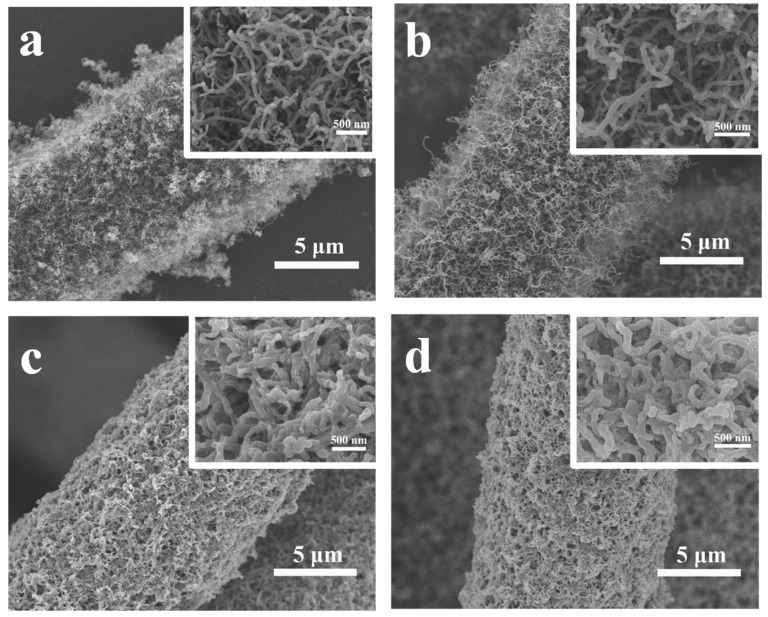
SEM images of (**a**) 3D-CNT, (**b**) 3D-NCNT, (**c**) 3D-CNT-Li_2_S_6_ composite and (**d**) 3D-NCNT-Li_2_S_6_ composite.

**Figure 3:**
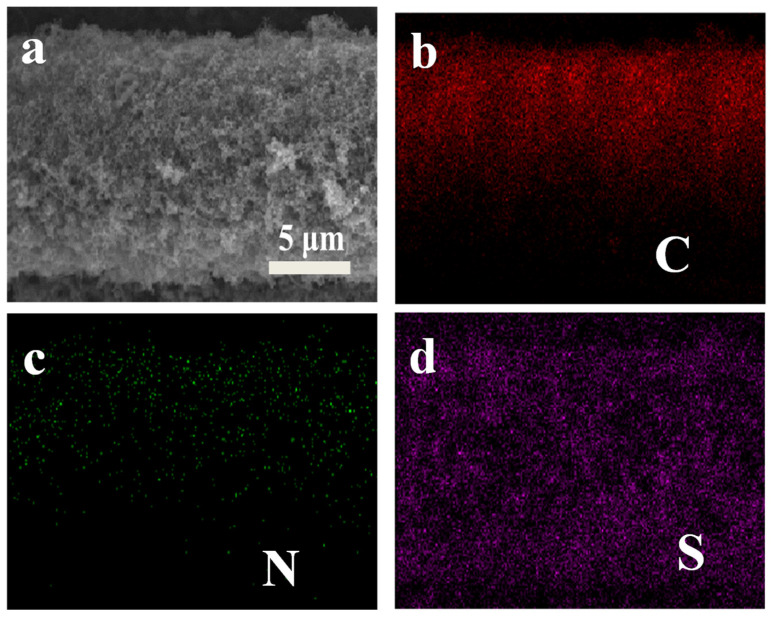
SEM image of (**a**) 3D-NCNT-Li_2_S_6_ composite and the corresponding EDS mapping for the element distribution of (**b**) C, (**c**) N, and (**d**) S.

The morphology of the samples at different stages is analyzed by SEM. As shown in Figure 2a, tens of the CNTs grow uniformly on the carbon fibers and construct a three dimensional conductive network, which ensures a fast ion/electron transportation [40,41]. The high-resolution SEM image shows that the interconnected CNTs with a diameter of ~50 nm provide a hierarchical microporous architecture. The sufficient void space is capable of loading a large amount of active material, accommodating the volumetric expansion of sulfur and maintaining high electrolyte absorbability [42]. The 3D-NCNT still maintain their microporous architecture as the 3D-CNT, demonstrating that nitrogen doping has no damage on the structure upon NH_3_ treatment (Figure 2b). Figure 2c,d present the SEM images of 3D-CNT-Li_2_S_6_ and 3D-NCNT-Li_2_S_6_ cathodes, the pores of the CNT and NCNT network are mostly filled with the active material. The whole structure of both cathodes is still porous, which allows efficient electrolyte penetration. It is obvious that the CNT and NCNT become thicker as they are coated by the active material and form a core-shell structure. The structure is further explored by the element mapping images of the EDS analysis (Figure 3). The elemental mapping evidently reveals the presence and homogeneous distribution of carbon, sulfur and nitrogen in the 3D-NCNT-Li_2_S_6_, suggesting that the nitrogen-doping has been doped into the CNT successfully and the Li_2_S_6_ uniformly cover the NCNTs [43].

**Figure 4:**
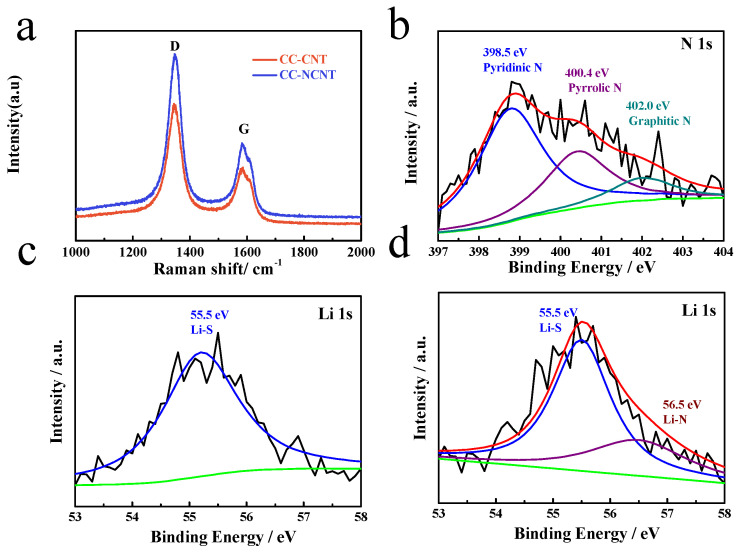
(**a**) Raman spectra of 3D-CNT and 3D-NCNT, XPS (**b**) N1s spectra of 3D-NCNT(**c**) Li1s spectra of 3D-CNT-Li_2_S_6_ and (**d**) 3D-NCNT-Li_2_S_6_ composite.

The variation of surface. chemistry of the samples is observed in Raman and X-ray photoelectron spectroscopy (XPS). As shown in Figure 4a, there are two broad peaks for 3D-CNT and 3D-NCNT at ~1350 cm^−1^ and 1587 cm^−1^, which can be attributed to strong graphitic G-band and weak disorder induced D-band, respectively [44,45]. The *I_D_*/*I_G_* ratio is an important parameter to evaluate the quality of graphic structure. The *I_D_*/*I_G_* ratio of 3D-NCNT is higher than that of 3D-CNT indicating that more defects exist in 3D-NCNT after NH_3_ treatment [44]. Figure 4b demonstrates the N1s spectra of 3D-NCNT, it can be deconvoluted into three peaks centered at 398.5, 400.2 and 402 eV, which are related to the pyridinic, pyrrolic and graphitic of N atoms form the nitrogen doping [46,47]. The nature of bonding between N atoms dopant on 3D-NCNT and polysulfide was analyzed. For 3D-CNT-Li_2_S_6_, only a peak at 55.5 eV corresponding to Li-S bond appears, implying no chemical bonding to 3D-CNT. Besides the Li-S bond, the 3D-NCNT-Li_2_S_6_ shows an additional peak at 56.5 eV that can be is assigned to Li–N bond, indicating chemical bonding between N atoms and polysulfide [48,49]. Previous work has proved that N dopants can increase the surface basicity of 3D-CNT, strengthening the Lewis acid-base interaction between 3D-NCNT and Lewis acidic Li in polysulfide [43,44].

### 3.2. Evaluation of Electrochemical Performance

**Figure 5:**
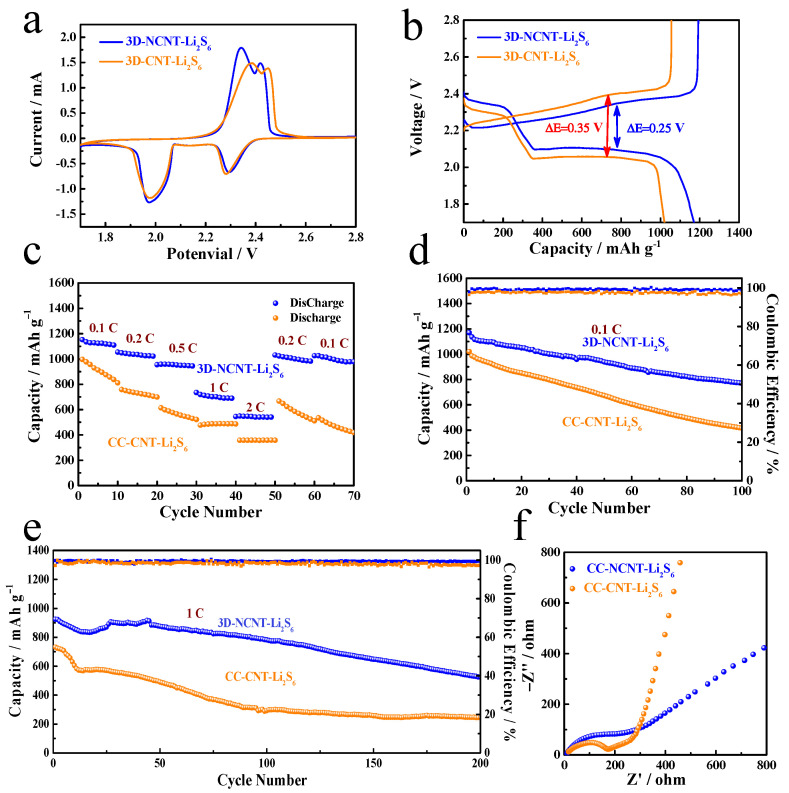
Comparison of electrochemical performance of 3D-CNT-Li_2_S_6_ and 3D-NCNT-Li_2_S_6_ cathodes, (**a**) CV curves at a scan rate of 0.1 mV s^−1^; (**b**) Typical voltage profiles at 0.1 C rate for first cycle; (**c**) Rate performance at current rates ranging from 0.1 C to 2 C; (**d**) Cycling performance at 0.1 C, (**e**) Cycling performance at 1 C; (**f**) Nyquist plots before cycling.

We utilized cyclic voltammetry (CV) to investigate the electrochemical reaction kinetics. Figure 5a displays the CV of 3D-CNT-Li_2_S_6_ and 3D-NCNT-Li_2_S_6_ in the voltage range from 1.7 to 2.8 V at the scan rate of 0.1 mV s^−1^, exhibiting the typical lithiation/delithiation features of sulfur cathodes [22,50]. The CV curve of 3D-CNT-Li_2_S_6_ and 3D-NCNT-Li_2_S_6_ both show two cathodic peaks in the reduction process, which can be attributed to the transformation of long chain Li_2_S_8_ to short chain lithium polysulfides and then to the insoluble Li_2_S_2_/Li_2_S, respectively. In the oxidization process, two anodic peaks are corresponding to reversible conversion from solid Li_2_S_2_/Li_2_S to short-chain lithium polysulfides and then to long-chain Li_2_S_8_, respectively [10,38]. Interestingly, the 3D-NCNT-Li_2_S_6_ cathode shows a distinguishable negative shift in the oxidation process and positive shift in the reduction process. Moreover, larger CV enclosed areas and higher peak intensities of the 3D-NCNT-Li_2_S_6_ cathode indicates a decrease of cell polarization and improved polysulfide redox kinetics [51], which is in good agreement with the galvanostatic charge/discharge profiles at a constant current rate of 0.1 C for the first cycle (Figure 5b). All the curves of 3D-CNT-Li_2_S_6_ and 3D-NCNT-Li_2_S_6_ cathodes demonstrate two typical plateaus of Li-S batteries during both the charge and discharge processes. Impressively, the 3D-NCNT-Li_2_S_6_ cathode exhibits a smaller voltage gap between charge and discharge plateaus, indicating a lower polarization and the enhanced reduction efficiency of lithium polysulfides via N doping [34,52]. The promotions on the 3D-NCNT-Li_2_S_6_ cathode can be further supported by electrochemical impedance spectroscopy (EIS) analysis (Figure 5f). The Nyquist plots of both cathodes consist of a single depressed semicircle in the high-medium frequency range and an inclined line in the low-frequency region, which are corresponding to the charge-transfer resistance (*R*_ct_) and Warburg impedance, respectively [53]. Compared with the semicircle and intercept on the *X*-axis in the Nyquist plots of both cathodes, the 3D-NCNT-Li_2_S_6_ cathode shows much lower charge-transfer resistance and Warburg impedance than that of the 3D-CNT-Li_2_S_6_ cathode. It reveals that N-doping reduces the inner resistance and improves the charge transfer at the electrode-electrolyte interface of the 3D-NCNT-Li_2_S_6_ cathode due to the strongest interaction between lithium polysulfides and NCNTs [28,34,39].

To evaluate the electrochemical stability of 3D-CNT-Li_2_S_6_ and 3D-NCNT-Li_2_S_6_ cathodes, the rate capability of both electrodes is tested at different currents from 0.1 to 2 C (Figure 5c). On cycling on the current densities of 0.1 C, 0.2 C, 0.5 C, 1 C, and 2 C, the 3D-NCNT-Li_2_S_6_ cathode exhibits excellent rate performance with the discharge capacities of 1158.7, 1054.1, 954.7, 735.1, and 545.6 mA h g^−1^ (the capacity is calculated based on the mass of sulfur), respectively. Even when the current density returns back from 2 to 0.2 C abruptly, the discharge capacity of 3D-NCNT-Li_2_S_6_ cathode is still recovered to 1031.3 mAh g^−1^, indicating good stability and robustness. As a contrast, the 3D-CNT-Li_2_S_6_ cathode reveals serious rapid capacity fading from 996.5 to 811.6 mA h g^−1^ at 0.1 C for the first 10 cycles and only delivers 357.9 mAh g^−1^ at 2 C. And after the current density is switched to 0.2 C, the capacity continues to descend.

**Figure 6:**
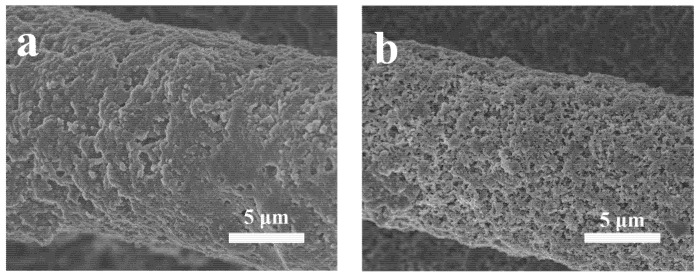
SEM of (**a**) 3D-CNT-Li_2_S_6_ composite and (**b**) 3D-NCNT-Li_2_S_6_ composite after cycling at 1 C for 200 cycles.

Moreover, long cycle performance of the 3D-CNT-Li_2_S_6_ and 3D-NCNT-Li_2_S_6_ cathodes is investigated. As shown in Figure 5d, it can be noted that the 3D-NCNT-Li_2_S_6_ cathode delivers a discharge capacity of 1170.8 mAh g^−1^ at 0.1 C, which is higher than that of the 3D-CNT-Li_2_S_6_ cathode (1020.4 mAh g^−1^), indicating higher utilization of active materials. After 100 cycles, a discharge capacity of 769.7 mAh g^−1^ is obtained for the 3D-NCNT-Li_2_S_6_ cathode, whereas the 3D-NCNT-Li_2_S_6_ cathode only delivers a lower discharge capacity of 415.3 mAh g^−1^. Additionally, the high-rate long cycling life of the 3D-CNT-Li_2_S_6_ and 3D-NCNT-Li_2_S_6_ cathodes is evaluated (Figure 5e). Under a current density of 1 C, the 3D-NCNT-Li_2_S_6_ cathode delivers a discharge capacity of 924.8 mAh g^−1^, and the discharge capacity can still maintain 525.1 mAh g^−1^ after 200 cycles. In contrast, the 3D-CNT-Li_2_S_6_ cathode only delivers low capacities and demonstrates fast capacity fading due to dissolution of the lithium polysulfides. Table 1 lists the performance of 3D-NCNT-Li_2_S_6_ with other CNT-S and N–doping graphene-sulfur cathodes reported in the literatures. All the sulfur cathodes with N-doping exhibit higher performance in terms of initial discharge capacity, rate capability and cycle life than that of sulfur cathodes without N-doping. The enhanced electrochemical performance can be ascribed to the N-doping. The graphitic nitrogen state can improve the conductivity of the carbon host, and the pyridinic nitrogen state can strongly attract lithium polysulfides with large enough binding energies to effectively anchor the soluble lithium polysulfides, due to an enhanced attraction between Li ions in lithium polysulfides and pyridinic nitrogen state and an additional attraction between S anions in lithium polysulfides and Li ions captured by the pyridinic nitrogen state [54]. Figure 6 demonstrates the SEM images of 3D-CNT-Li_2_S_6_ and 3D-NCNT-Li_2_S_6_ cathodes after 200 cycles. And the morphological change provides direct evidence for suppressing the lithium polysulfide dissolution by chemical absorption. Note that the 3D-NCNT-Li_2_S_6_ cathode after 200 cycles still remains the similar structure to that of the cathode before cycling, while the 3D-CNT-Li_2_S_6_ cathode becomes unrecognizable, with serious aggregation. The chemisorption of lithium polysulfides on NCNT render a uniform re-deposition of sulfur or Li_2_S during the charge and discharge process in conductive CNT network that gives rise to more stable and higher ionic and electronic conductivity of the 3D-NCNT-Li_2_S_6_ cathode. As N doping in the CNTs network forms the chemical bond between Li and N that can effectively confine the lithium polysulfides among the networks, this improves the electrochemical reaction kinetics as well as enables the network catalyze the redox reactions to reduce polarization [29,41,44].

## 4. Conclusions

In summary, we developed a novel strategy for construction of a three-dimensional N-doped CNTs networks by using CVD technology and NH_3_ treatment which supports lithium polysulfides as a binder-free cathode for high-performance Li-S batteries. The three-dimensional N-doped CNTs networks not only construct a conductive porous 3D architecture for facilitating fast ion and electron transport, but they also create large amounts of void spaces and porous channels as well, for a high sulfur loading with 3.84 mg cm^−2^. In addition, lithium polysulfides can be effectively confined among the networks through the chemical bond between Li and N. Moreover, N doping in the CNTs network plays an important role in improving the electrochemical reaction kinetics as well as enabling the network to catalyze the redox reactions to reduce polarization. As a result, significantly enhanced charge capacity and cyclic stability have been achieved for the 3D-NCNT-Li_2_S_6_ cathode. Therefore, our research may provide a facile and scalable way to design a binder-free lithium polysulfides based cathode for Li-S batteries in the future.

## Figures and Tables

**Table 1:** Comparison of electrochemical performances of CNT-S and N-doping graphene-sulfur electrodes.

Cathode	Rate	Initial Discharge Capacity (mAh g^−1^)	Stable Discharge Capacity (mAh g^−1^) and Cycles
S-CNT [55]	0.1C	1109	740 after 100
PCNT-S [56]	0.1C	895	625 after 100
CNT-S [57]	0.1C	736.8	408.4 after 85
S-MWCTs [58]	100 mA/g	1330	854 after 30
CNT/S [59]	0.1C	864	358 after 100
A-3DNG/S [60]	0.2C	1101	860 after 200
N-G-S [54]	0.3A/g	1150	880 after 100
S@N-3D-rGO [61]	0.2C	1042	987 after 100
3DNG-S [62]	0.2C	1050	990 after 100
3D-NCNT-Li_2_S_6_ (This work)	0.1C	1170.8	769.7 after 100

## Data Availability

The data presented in this study are available on request from the corresponding author.
